# *Bacillus cereus* isolated from a positive bone tissue culture in a patient with osteolysis and high-titer anti-interferon-γ autoantibodies: A case report

**DOI:** 10.1097/MD.0000000000017609

**Published:** 2019-10-25

**Authors:** Ye Qiu, Jianquan Zhang, Bixun Li, Hong Shu

**Affiliations:** a Department of Comprehensive Internal Medicine, the Affiliated Tumor Hospital of Guangxi Medical University,; b Department of Respiratory Medicine, The First Affiliated Hospital of Guangxi Medical University,; c Microbiology Laboratory, the Affiliated Tumor Hospital of Guangxi Medical University, Nanning, Guangxi, China.

**Keywords:** anti-IFN-γ autoantibodies, *B cereus*, osteolysis

## Abstract

**Rationale:**

*Bacillus cereus* (*B cereus*) is an aerobic or facultative anaerobic gram-positive, spore-forming bacterium. It can cause fatal disease and generally manifests as 3 distinct syndromes: food intoxication, localized infection, and systemic infection. It is a rare infection that can occur in immunocompetent persons with osteolytic and high-titer anti-IFN-γ autoantibodies.

**Patient concerns:**

We reported a case of an HIV-negative 24-year old man with an interrupted fever and a 20-day history of progressive ache in the right thigh and high-titer anti-IFN-γ autoantibodies. Magnetic resonance imaging, X-radiography, high-resolution computed tomography, and 3-dimensional reconstruction of the bone showed multiple lucent defects with moth-eaten destruction of the bone and cortical substance of bone in the right femur. Emission CT showed significantly increased uptake in the femur.

**Diagnosis and Interventions:**

The patient was originally misdiagnosed with osteosarcoma; acute osteomyelitis was also considered. He received intravenous piperacillin, sulbactam, and levofloxacin during hospitalization; however, he did not respond to the 3-week antibiotic course and his condition worsened. After cultures from incisional biopsy specimens were obtained from the femoral cavity, *B cereus*-induced osteomyelitis was diagnosed. He received intravenous injections of moxifloxacin 400 mg qd for 4 weeks and oral moxifloxacin 400 mg qd for 8 weeks.

**Outcomes:**

The patient's symptoms and signs improved. His X-radiography, HRCT, MRI, and 3-dimensional reconstruction of the bone showed absolute absorption in the right femur. However, the anti-IFN-γ autoantibody titer was still high. No recurrence was observed after 24 months of follow-up. He is still undergoing follow-up at this time.

**Lessons:**

This is the first case involving a patient with *B cereus* infection showing a high titer of anti-IFN-γ autoantibodies. *B cereus* infection can involve the bone, leading to osteolysis in HIV-negative individuals. Although this patient was HIV-negative and had no other comorbidities, the presence of high titer anti-IFN-γ autoantibodies may be the primary reason for *B cereus* infection. Clinicians should pay more attention to the identification of osteolytic destruction caused by tumor and infection.

## 1 Introduction

Production of anti-interferon (IFN)-γ autoantibodies has recently been recognized as a mechanism in nontuberculous mycobacteria (NTM) infection. The autoantibody against IFN-γ has also been recognized as a cause of both adult-onset immunodeficiency and a risk factor for infections due to opportunistic pathogens, including *Cryptococcus neoformans*, *Histoplasma capsulatum*, *Burkholderia* spp, *Talaromyces (Penicillium) marneffei*, and disseminated salmonellosis, especially in Asian patients.^[1,2]^ However, anti-IFN-γ autoantibodies have not previously been associated with *Bacillus cereus* infections in the absence of other opportunistic infections.

*B cereus* is an aerobic or facultative anaerobic gram-positive, spore-forming bacterium. Its natural reservoir includes soil, decaying organic matter, marine water, the intestinal tract of invertebrates, vegetables, and other common foods, and the spore is refractory to extreme environmental conditions, such as alcohol-based hand-washing products, pasteurization, or γ-radiation.^[3–4]^*B cereus* can also cause fatal disease and generally manifests in three distinct syndromes: food intoxication, localized infection, and systemic infection. Bacteremia, pneumonia, meningitis, brain abscess, endophthalmitis, skin and soft-tissue infections, pyelonephritis, and endocarditis due to *B cereus* have been reported in hospital settings.^[4]^ While cases of acute osteomyelitis and osteolysis have been rare, they have been reported to occur in immunocompromised patients in tropical and subtropical regions. The reason for this vulnerability is still not yet clear. Herein, we report a case of an HIV-negative 24-year old man with a high anti-IFN-γ autoantibody titer, which was considered to play an important role in an opportunistic pathogenic infection caused by *B cereus.*^[5,6]^

## 2 Case presentation

**Figure 1:**
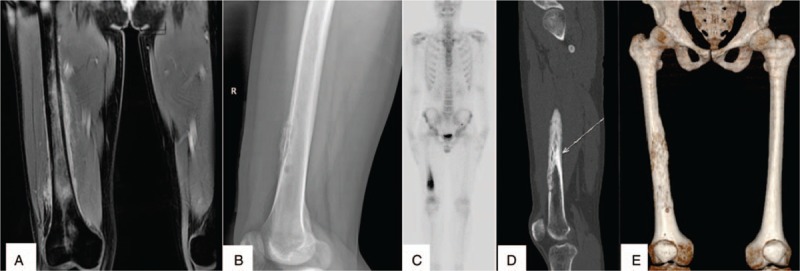
X-radiography, high-resolution computed tomography, and 3-dimensional reconstruction of the bone showing multiple lucent defects with a moth-eaten destruction of osteolysis in the right femur (A–C). Emission CT showed significantly increased uptake in the femur (D). Magnetic resonance imaging shows abnormal signal shadow in the femoral bone marrow cavity (E).

**Figure 2:**
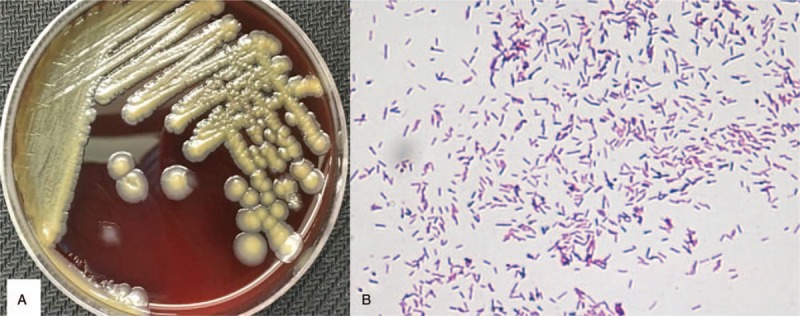
Cultures from the femoral cavity lesions grew *Bacillus cereus* rods (A). Gram stain of the femoral bone marrow cavity lesion culture showing gram-positive slender bacilli with rounded ends singly, in pairs, and in short chains (×400) (B).

**Figure 3:**
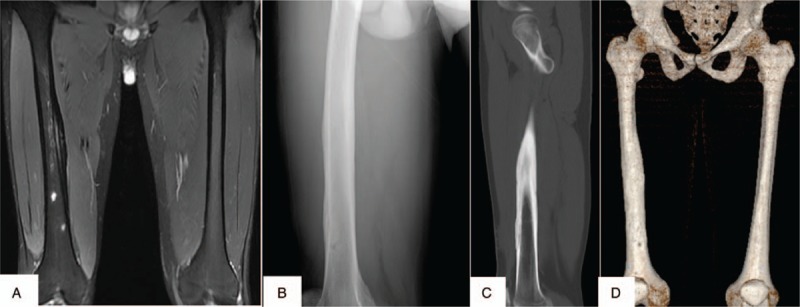
X-radiography, high-resolution computed tomography, magnetic resonance imaging, and 3-dimensional reconstruction of the bone showing absolute absorption in the right femur (A–D).

An immunocompetent 24-year-old man from southern China presented to the internal medicine department with an interrupted fever and a 20-day history of a progressive ache in the right thigh. He reported having developed a parulis 2 months ago. Physical examination revealed a lump located in the right lower third of the thigh. Routine blood examination revealed a leukocyte count of 16.30 × 10^9^/L and neutrophil percentage of 93.2%. His CD4 T-cell count was 426/μL, C-reactive protein level was 38.9 mg/L, and erythrocyte sedimentation rate was 100.4 mm/hour. His serum anti-IFN-γ autoantibody titer was 1053.895 ng/mL, which was elevated. He was negative for antistreptolysin O, antinuclear antibody, rheumatoid factor, and HIV from blood sample tests. Magnetic resonance imaging (MRI) showed an abnormal signal shadow in the femoral bone marrow cavity (Fig. 1A). X-radiography (Fig. 1B), high-resolution computed tomography (HRCT) (Fig. 1D), and 3-dimensional reconstruction of the bone (Fig. 1E) showed multiple lucent defects with the moth-eaten destruction of the bone and cortical substance of bone in the right femur. Emission CT (ECT) showed significantly increased uptake in the femur (Fig. 1C). Acute osteomyelitis or primary femoral tumor was considered. The patient received intravenous piperacillin, sulbactam, and levofloxacin during hospitalization, but did not respond to the 3-week antibiotic course and his condition worsened. X-ray, HRCT, 3-dimensional reconstruction of bone, ECT, and MRI of right thigh showed worsened leg lesions. Histopathological examination of incisional biopsy specimens obtained from the lower third of the femoral cavity revealed infiltration of a few leukomonocytes; however, no tumors or pathogens were identified. Cultures from femoral cavity lesions grew *B cereus* rods (Fig. 2A and B), the identity of which was confirmed by VITEKMS. Acute *B cereus-*induced osteomyelitis was diagnosed. The patient received intravenous injections of moxifloxacin 400 mg qd for 4 weeks and oral moxifloxacin 400 mg qd for 8 weeks. With this treatment, the symptoms and signs improved. His X-radiography, HRCT, MRI, and 3-dimensional reconstruction of the bone showed absolute absorption in the right femur (Fig. 3A–D). The level of anti-IFN-γ autoantibodies was still high, with a titer of 1293.032 ng/ml. However, no recurrence was observed after 24 months of follow-up. He is still undergoing follow-up at this time.

## 3 Discussion and conclusions

*B cereus* is an aerobic or facultative anaerobic gram-positive, spore-forming bacterium that exists ubiquitously in soil, marine environments, vegetables, the intestinal tracts of invertebrates, and the human skin.^[3,4]^ It manifests in 3 distinct syndromes: food intoxication, localized, and systemic infections.^[3]^ Several organs and tissues have been described as possible targets of *B cereus*, such as the lung, eye, liver, soft-tissue and brain, leading to diseases, such as bacteremia and meningitis, especially among immunocompromised patients who have central venous catheters.^[3–4]^ However, to date, cases of acute osteomyelitis and osteolysis have been rare and only reported in immunocompromised patients in tropical and subtropical regions. Recently, a study showed that osteolysis can be found in HIV-negative individuals with disseminated talaromycosis.^[5]^ To differentiate *B cereus* infections from tumors and talaromycosis, lesion biopsies and cultures for pathologic examination are efficacious and pivotal.

Recently, several studies recognized anti-IFN-γ autoantibodies as a cause of adult-onset immunodeficiency and a risk factor for infections due to opportunistic pathogens, including *C neoformans*, *H capsulatum*, *B spp*, *T (Penicillium) marneffei*, and disseminated salmonellosis, especially in Asian patients.^[1,2,5]^ Our patient did not have HIV or other underlying diseases; however, his titer of anti-IFN-γ autoantibodies was high. High-titer anti-IFN-γ autoantibodies can inhibit interleukin (IL)-12 production. Disruption of the IL-12-dependent interferon-gamma (IFN-γ) axis, which is the main regulatory pathway of cell-mediated immunity, may lead to immune system defects; in addition, this axis may play a critically important role in providing protection against these intracellular organisms.^[1,6]^ Thus, the patient's high-titer anti-IFN-γ autoantibodies may lead to immune system defects, which increase the risk of *B cereus* infection. The clinical manifestations of patients with *B cereus*, such as necrotizing infections, might be caused by the release of exotoxins, such as proteases, phospholipases, and hemolysins.^[4]^ Inflammation often leads to tissue remodeling and bone resorption, processes that are subject to inhibition by IFN-γ.^[7–9]^ Bone resorption is mediated by myeloid lineage cells called osteoclasts, and IFN- γ is a potent inhibitor of osteoclastogenesis. Several studies have shown that osteoclast formation and bone destruction were more pronounced in mice lacking functional IFN-γ.^[8–10]^ IFN-γ induces rapid degradation of the RANK adaptor protein and tumor necrosis factor receptor-associated factor 6, which results in strong inhibition of the RANKL-induced activation of the transcription factor NF-kB and c-Jun N-terminal kinase.^[7]^ Under conditions of high-titer anti-IFN-γ autoantibodies, IFN-γ can be neutralized and incapacitated, and the net balance of these opposing forces is biased towards osteolysis.

Regarding treatment, there is a lack of recommended standardized treatment options for patients with *B cereus* infections. In previous studies, we found varying antimicrobial regimens, mostly used for a duration of 6 weeks.^[3,8–11]^ According to antimicrobial susceptibility studies, *B cereus* appears to be uniformly sensitive to gentamicin, imipenem, and vancomycin.^[8–11]^ Most strains were variably resistant to amoxicillin (40%), cefazolin (55%), ceftriaxone (40%), ciprofloxacin (41%), clindamycin (20%), and penicillin (100%).^[8–10]^ A retrospective single-center trial involving 29 evaluable patients showed that no significant difference existed in the clinical responses of the 2 groups in terms of all-cause mortality; however, early defervescence occurred more often with appropriate empirical therapy than with inappropriate empirical antimicrobial therapy.^[3]^ This study also reported that 65.5% of isolates were resistant to clindamycin and 10.3% were resistant to levofloxacin. The patient reported in this current case was empirically treated with piperacillin, sulbactam, and levofloxacin during hospitalization; however, he did not respond and his condition worsened. This suggested that the organism was resistant to these antimicrobials. After changing the regimen to moxifloxacin 400 mg qd for 12 weeks, the symptoms and signs drastically improved. No relapse was observed during the 24-month follow-up during which he stopped the oral moxifloxacin treatment.

## Author contributions

**Data curation:** Ye Qiu, Hong Shu.

**Formal analysis:** Jianquan Zhang.

**Funding acquisition:** Ye Qiu.

**Investigation:** Ye Qiu, Jianquan Zhang.

**Methodology:** Ye Qiu, Jianquan Zhang.

**Project administration:** BiXun Li.

**Resources:** Ye Qiu, Hong Shu.

**Software:** Hong Shu.

**Writing – original draft:** Ye Qiu.

**Writing – review & editing:** Jianquan Zhang, BiXun Li.
